# Re: Farmers' suicides in the Vidarbha region of Maharashtra, India: a qualitative exploration of their causes

**DOI:** 10.5249/jivr.v6i1.471

**Published:** 2014-01

**Authors:** Amit Agrawal

**Affiliations:** ^a^Department of Neurosurgery, Narayana Medical College Hospital, Chinthareddypalem, Nellore, Andhra Pradesh, India.

The article “Farmers' suicides in the Vidarbha region of Maharashtra, India: a qualitative exploration of their causes” discusses in details the reasons, common factors, and solutions for farmers' suicides in the Vidarbha region of Maharashtra.^1^ It should make us think that in spite of being the main focus of any development plan of India, by each passing year the agriculture as an industry is loosing its importance for policy makers resulting in severe distress among the farmers leading to recent dramatic rise in the number of suicides.^2^ Up to 40% farmers opted to quit agriculture in favor to take up some other carrier^2^ is a matter of concern. Previously published work form the Vidarbha region also the involvement and role of medical professionals in assessing and finding out the solutions of our current social problems and to address the problem on an urgent basis.^2^ However the detail interview findings of verbal autopsy were not available and further discussed in the study. Dongre et al^1^ not only provide more details account of underlying causes of Farmers’ suicide but also propose potential solution for the same. For example chronic pesticide exposure not only is found to be associated with suicidal ideation, but also a high level of pesticide exposure can be associated the high suicide risk.^3^ Also studies have highlighted the farmers concerns for chemical fertilizers and environmental degradation as their repeated degradation can result in the loss of land productivity thus putting future generations of farmers at even greater risk.^4^ It has been well suggested that there is a need for the promotion of organic farming and not only reducing dependency on chemical fertilizers and pesticides^3^ but also avoiding high level of pesticide exposure and high suicide risk.^1^ It is vital that these messages are disseminated as widely as possible to the appropriate policy-makers.^3^ We all know that in agriculture there is no surety of crop success, no fixed income and in addition there may be unexpected natural calamities. For the farmers, if they are producing more grains like many other fields there are no felicitations, rewards or recognition. We all would be agreeing that developing socio-economic zones and creating more and more jobs most of the time will provide the fixed income. In such a scenario how it will be possible for us to convince peoples to continue with the highly vulnerable profession like agriculture as their livelihood. Though religious leaders have a major role to play in suicide prevention^2^ but we believe that farmer friendly economic reforms strategies, percolating till the needy will have the major impact. There is need for well designed field-based prospective studies to understand all the issues in details and to find out comprehensive intervention strategies to offer support and counseling to vulnerable farmers in rural areas.^1^
